# Supplementary material for: Uncovering drone intentions using control physics informed machine learning
Source: Commun Eng. 2024 Feb 24;3:36. doi: 10.1038/s44172-024-00179-3 (PMC11053008; doi:10.1038/s44172-024-00179-3)
Supplement: Supplementary file 2 — Supplementary Material [file 44172_2024_179_MOESM2_ESM.pdf]

Supplementary Material for:
Uncovering Drone Intentions using Control Physics Informed Machine Learning

Adolfo Perrusquía, Weisi Guo, Benjamin Fraser, Zhuangkun Wei

School of Aerospace, Transport and Manufacturing, Cranfield University MK43 0AL, Bedford, UK. e-mail: adolfo.perrusquia-guzman@cranfield.ac.uk

Supplementary Note 1 Preliminaries

This supplementary document provides a detailed description of the proposed algorithms, data generation, additional results, theoretical results that include stability and convergence proofs for uncovering drone intentions using control physics informed machine learning.

Supplementary Table 1: **Models used throughout this research paper**

Acronym	Description
CPhy-ML	Control-Physics Informed Machine Learning
EKF	Extended Kalman Filter
RNN	Recurrent Neural Network
LSTM	Long Short Term Memory
GRU	Gated Recurrent Unit
CBLSTM	Convolutional Bidirectional Long Short Term Memory
CBLSTMA	Convolutional Bidirectional Long Short Term Memory with Attention layer
CNN	Convolutional Neural Network
CNNA	Convolutional Neural Network with Attention
DMoE	Deep Mixture of Experts
RC Linear	Reservoir Computing with Linear Decoder
RC SVM	Reservoir Computing with Support Vector Machine Decoder
RC MLP	Reservoir Computing with Multi-Layer Perceptron Decoder
PIRC	Physics Informed Reservoir Computing
DMDc	Dynamic Mode Decomposition with Control.
LQR	Linear Quadratic Regulator
DMD-LQR	Dynamic Mode Decomposition with Linear Quadratic Regulator control
DARE	Discrete Algebraic Riccati Equation
ARE (CARE)	(Continuous) Algebraic Riccati Equation
IRL	Inverse Reinforcement Learning

Supplementary Note 1.1 Notations

Throughout this material, $\mathbb{N}$, $\mathbb{R}$, $\mathbb{R}^n$, $\mathbb{R}^{n\times m}$ denote the spaces of natural numbers, real numbers, real n-vectors, and real $n \times m$-matrices, respectively; $\boldsymbol{I}_n \in \mathbb{R}^{n\times n}$ denotes an identity matrix of $n \times n$; $\lambda_{\min}(\boldsymbol{A})$ and $\lambda_{\max}(\boldsymbol{A})$ denote the minimum and maximum eigenvalues of matrix $\boldsymbol{A}$, $\otimes$ denotes the Kronecker product, $\mathrm{vec}(\boldsymbol{A})$ is the vectorization of matrix $\boldsymbol{A}$, $\mathrm{mat}(\boldsymbol{x})$ is the matrization of the vector

$\boldsymbol{x}$, the norms $\|\boldsymbol{x}\|_2 = \sqrt{\boldsymbol{x}^\top \boldsymbol{x}}$ and $\|\boldsymbol{X}\|_\mathcal{F} = \sqrt{\mathrm{tr}\{\boldsymbol{X}^\top \boldsymbol{X}\}}$ stand for the Euclidean and Frobenius norms, respectively; $\mathrm{tr}\{\cdot\}$ defines the trace function, where $x \in \mathbb{R}$ is a scalar, $\boldsymbol{x} \in \mathbb{R}^n$ is a vector, and $\boldsymbol{X} \in \mathbb{R}^{n\times m}$ is a matrix with $n, m \in \mathbb{N}$.

Supplementary Note 1.2 Activation functions and Metrics

The activation functions and metrics used in this work are summarized in Supplementary Tables 1 and 2.

Supplementary Table 2: **Activation functions and Metrics used in this research**

Acronym	Description
Tanh	Hyperbolic Tangent Function
RBF	Radial Basis Function
ReLU	Rectified Linear Unit
MSE	Mean Squared Error
Recon MSE	Mean Squared Reconstruction Error
MAE	Mean Absolute Error
RMSE	Root Mean Squared Error
RMSSNE	Root Mean Squared Spectral Norm Error
R^2	Coefficient of determination

Supplementary Note 2 Synthetic Data Generation

The experiments are conducted using a XPS Laptop endowed with NVIDIA GeForce RTX 2060 with Max-Q Design. Python 3.9.0 and Matlab 2022b are used as main programming softwares.

Supplementary Note 2.1 Simulated Radar Tracks

The data used in this research is generated from telemetry data obtained from open-access sources [1–4]. These data define four trajectory intention classes: package delivery, point-to-point flights, mapping flights and perimeter flights.

We use the longitude, latitude, altitude, lateral velocity, longitudinal velocity,and vertical velocity. The longitude, latitude and altitude are converted into Cartesian positions. We create synthetic data from the combined datasets. This is achieved by using the Stone Soup software [5] to simulate radar measurements by modifying the location of a virtual radar and the amount of noise in the measurement model. Extended Kalman filter is used to obtain the tracks of the new simulated radar measurements.

Fig. 1 shows data generated from a single sample trajectory from the perimeter flight dataset. In this example, we use the following locations for the virtual radar: $(0, 0)$, $(250, 0)$, $(0, 250)$, and $(500, 500)$. We apply a change of coordinates to the input trajectories such that the virtual radar is always located at the origin $(0, 0)$. From the results we can observe that different radar measurements are obtained by only modifying the location of the virtual radar. In consequence, different trajectories are obtained from the extended Kalman filter which are useful to construct a rich and heterogeneous dataset to train and test the models developed in this research.

Supplementary Note 2.2 Simulated RF data

We use telemetry data collected from personal use drone and simulated data to emulate radio frequency (RF) data [6] which contains information of the control input. The control input contains information of the Euler angles: roll ϕ, pitch θ and yaw ψ, and the total thrust μ. Here, the trajectories are corrupted by Gaussian noise weighted by the standard deviation of each input feature. Fig. 2 shows some trajectory profiles and control input values used in the research. Each experiment lasts 2 minutes and the trajectories are sampled each 0.02 seconds.

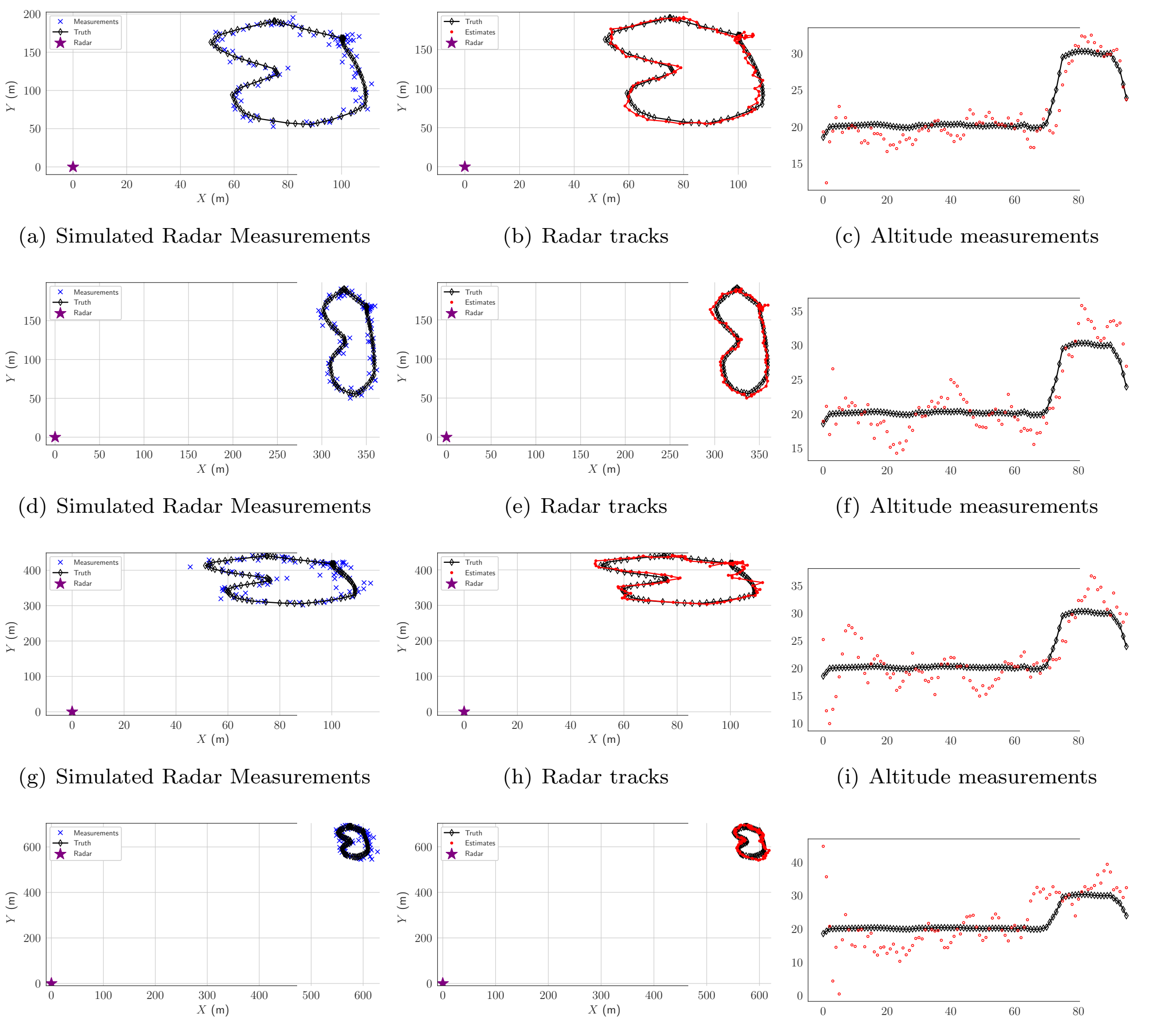

(a) Simulated Radar Measurements (b) Radar tracks (c) Altitude measurements

(d) Simulated Radar Measurements (e) Radar tracks (f) Altitude measurements

(g) Simulated Radar Measurements (h) Radar tracks (i) Altitude measurements

(j) Simulated Radar Measurements (k) Radar tracks (l) Altitude measurements

Supplementary Figure 1: **Simulated Radar Tracks under different virtual radar locations**. Ground truth are plotted with black lines with diamond markers, measurements are plotted with cross mark, estimated tracks are plotted with red dots, and radar location is plotted with a violet star. Each row represents the estimated radar tracking results when the radar is located at $(0, 0)$, $(250, 0)$, $(0, 250)$ and $(500, 500)$, respectively.

Supplementary Note 3 Hybrid Classifier Additional Results

The hybrid classifier is composed by a CBLSTMA [7,8] classifier and a LSTM autoencoder [9] and it is given by the green block of Fig. 3. Here the input data trajectories are divided in sub-trajectories in accordance with a pre-defined window length. In this research, we use four different window lengths to test the prediction capabilities of the approach. The window lengths used in this research are: 8, 16, 32, and 64. This step is important to ensure the algorithm works in real-time.

Supplementary Note 3.1 Hyperparameters

The hyperparameters used in the trajectory intention classifier are summarized in Supplementary Table 3.

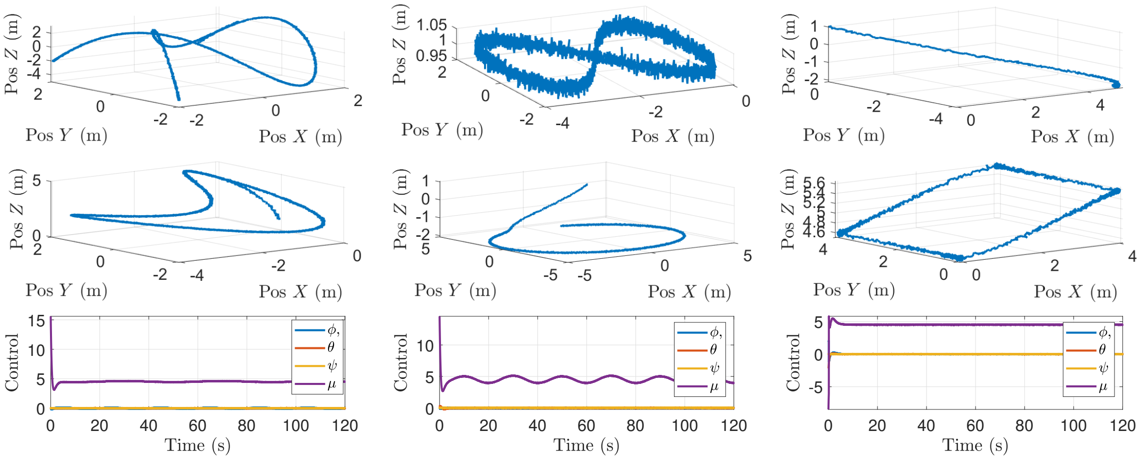

Supplementary Figure 2: **Real-world data sample trajectories**. Telemetry data is plotted with blue solid line, control signals are plotted in blue, rd, yellow and purple solid lines. The telemetry data are corrupted by noise to emulate real RF measurements. The final row show examples of the control input measurements.

Supplementary Table 3: **Hyperparameters of the Trajectory intention classifiers**.

Parameter	Deep Learning (DL) models								
	LSTM		GRU		CBLSTM/CBLSTMA			CNN/CNNA	
	Dense	RNN	Dense	RNN	Dense	RNN	CNN	Dense	CNN
Activations	ReLU	Tanh	ReLU	Tanh	ReLU	Tanh	ReLU	ReLU	ReLU
Hidden units	20	20	20	20	64	20	20	64	[32,64]
Dropout	-	0.4	-	0.4	0.3	0.3	-	-	0.3
Epochs	75		75		50			50	
Batch size	256		256		128			128	
Optimiser	Adam		Adam		Adam			Adam	
Learning rate	1e-3		1e-3		1e-3			1e-3	
Loss	Cat. Cross Entropy		Cat. Cross Entropy		Cat. Cross Entropy			Cat. Cross Entropy	

Supplementary Note 3.2 Extended Results

Classification results across each window length are summarized in Supplementary Table 4. Anomaly detection results across all time-windows are summarized in Supplementary Table 5.

The confusion matrix results across all window lengths are shown in Fig. 4. From these matrices we observe that small window lengths tend to have more false positives and negatives in comparison with larger window lengths that possesses more information about the mission profile.

The accuracy, categorical cross-entropy and reconstruction error losses per window length are given in Fig. 5. The results show that the classifier tends to overfit the training data. Therefore, we use early stopper as regularisation mechanism to stop the learning phase when there is no improvement in the validation curve after certain number of epochs. Conversely to the classifier results, the novelty detector works better with small window lengths, whilst for larger ones it has more reconstruction error. This result was expected because large window lengths imply that the trajectories have more variability and, in consequence, it is hard for each expert to perfectly reconstruct their respective trajectory classes.

Fig. 6 shows some extended results of the trajectory intention classifier in some random examples of the testing dataset. The results show high accurate prediction results, whilst the reconstruction error is small because the trajectories matches with the trajectory intention classes of the training data.

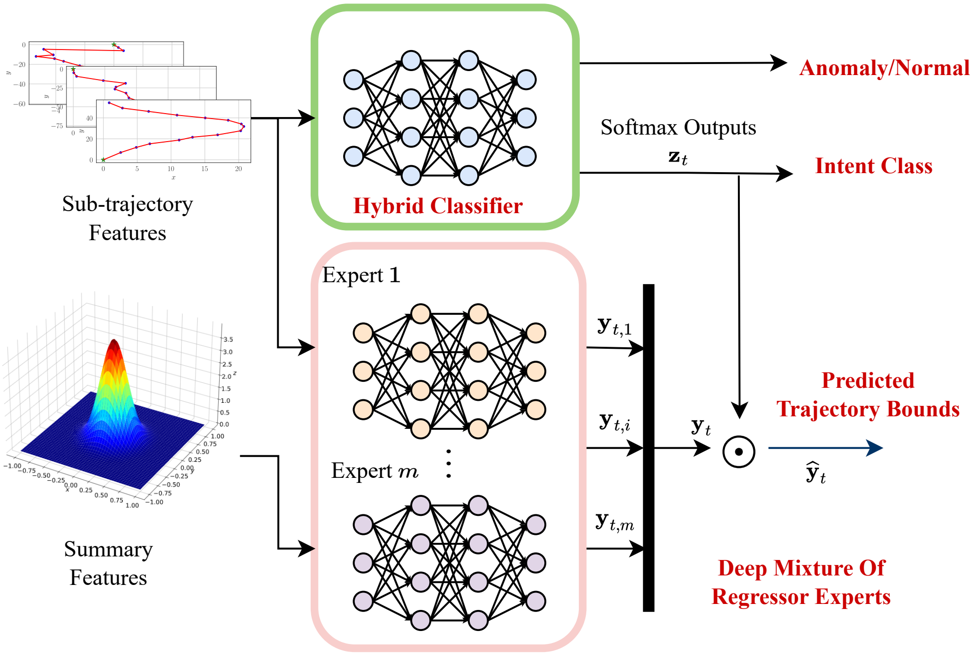

Supplementary Figure 3: **Hybrid Classifier & Novelty Detection Architecture**. Composed by a hybrid classifier and a deep mixture of experts networks.

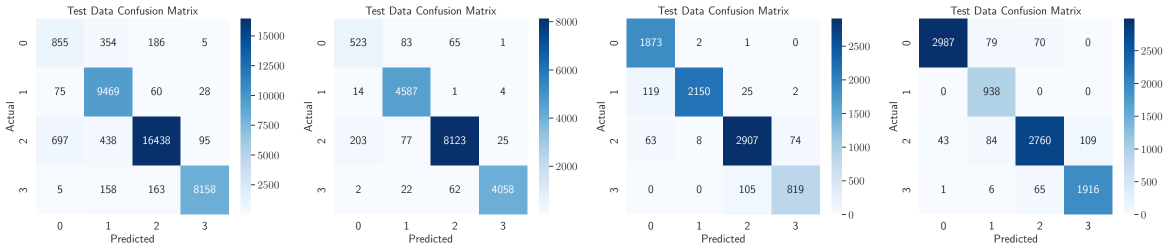

(a) Window length 8 (b) Window length 16 (c) Window length 32 (d) Window length 64

Supplementary Figure 4: **Confusion matrix of the Hybrid Classifier.** Results in the testing dataset

Supplementary Note 4 Trajectory Intent Regression

The trajectory intent regression architecture is given by the pink block of Fig. 3. This architecture has as input the sub-trajectory features and in addition summary features composed by the mean, standard deviation, maximum and minimum values of each sub-trajectory feature. The architecture is based on a deep mixture of experts, where each expert is a multi-input convolutional neural network [10] trained on each trajectory intention class.

Supplementary Note 4.1 Hyperparameters

Th hyperparameters used in the trajectory intention regression models are summarized in Supplementary Table 6.

Supplementary Table 4: **Trajectory intention classification results.**

Model	Window Length (s)	Validation				Test			
		Accuracy	Precision	Recall	F1-Score	Accuracy	Precision	Recall	F1-Score
Random Forest	8	0.8667	0.7953	0.7414	0.7545	0.8920	0.9006	0.8106	0.8340
LSTM		0.8282	0.7215	0.6972	0.7046	0.9146	0.9128	0.9145	0.9136
GRU		0.8701	0.8191	0.7790	0.7949	0.8854	0.9037	0.8028	0.8313
CBLSTM		0.9133	0.8704	0.8614	0.8656	0.9636	0.9570	0.9686	0.9625
CBLSTMA		**0.9166**	**0.8744**	**0.8888**	**0.8812**	**0.9683**	**0.9632**	**0.9722**	**0.9675**
CNN		0.8983	0.8523	0.8789	0.8644	0.9424	0.9299	0.9482	0.9386
CNNA		0.9156	0.8723	0.8724	0.8723	0.9626	0.9542	0.9662	0.9599
Random Forest	16	0.8730	0.7978	0.7457	0.7556	0.8973	0.9133	0.8179	0.8416
LSTM		0.9028	0.8449	0.8674	0.8554	0.9621	0.9561	0.9657	0.9607
GRU		0.9305	0.8985	0.8980	0.8982	0.9700	0.9658	0.9715	0.9684
CBLSTM		0.9416	**0.9103**	0.9096	0.9098	0.9794	0.9755	**0.9843**	0.9796
CBLSTMA		**0.9433**	0.9087	**0.9200**	**0.9141**	**0.9798**	**0.9760**	0.9840	**0.9798**
CNN		0.9395	0.9030	0.9139	0.9083	0.9765	0.9726	0.9794	0.9757
CNNA		0.9388	0.9015	0.9175	0.9091	0.9760	0.9719	0.9795	0.9755
Random Forest	32	0.8671	0.7746	0.7367	0.7406	0.9336	0.9328	0.9355	0.9332
LSTM		0.9486	0.9232	0.9421	0.9322	0.9673	0.9600	0.9733	0.9680
GRU		0.9515	0.9314	0.9307	0.9305	0.9758	0.9686	0.9833	0.9755
CBLSTM		0.9466	0.9089	0.9206	0.9141	0.9842	0.9814	0.9888	0.9850
CBLSTMA		**0.9548**	**0.9327**	**0.9330**	**0.9324**	**0.9855**	**0.9828**	**0.9897**	**0.9861**
CNN		0.9431	0.9080	0.9068	0.9067	0.9799	0.9762	0.9854	0.9805
CNNA		0.9513	0.9221	0.9171	0.9192	0.9817	0.9784	0.9858	0.9819
Random Forest	64	0.8957	0.8456	0.7731	0.7852	0.9592	0.9578	0.9730	0.9645
LSTM		0.9623	0.9596	0.9376	0.9472	**0.9923**	**0.9910**	**0.9946**	**0.9928**
GRU		0.9673	**0.9673**	0.9393	0.9515	0.9903	0.9888	**0.9946**	0.9916
CBLSTM		0.9666	0.9545	0.9478	0.9508	0.9830	0.9811	0.9911	0.9858
CBLSTMA		**0.9720**	0.9663	0.9503	**0.9576**	0.9843	0.9825	0.9916	0.9868
CNN		0.9668	0.9403	0.9513	0.9455	0.9773	0.9745	0.9879	0.9808
CNNA		0.9690	0.9590	**0.9524**	0.9554	0.9823	0.9811	0.9903	0.9854

[1] Best results are in bold.

Supplementary Table 5: **Extended Hybrid Classifier & Anomaly Detector final results.**

Window Length	Training	Validation					Test					Other (anomalous flights)
	Recon MSE	Accuracy	Precision	Recall	F1 Score	Recon MSE	Accuracy	Precision	Recall	F1 Score	Recon MSE	Recon MSE
8	0.1801	0.9217	0.8867	0.8953	0.8906	1.0160	0.9591	0.9523	0.9637	0.9577	0.4308	4748758.0000
16	0.1909	0.9359	0.9014	0.9005	0.9009	1.4313	0.9757	0.9714	0.9808	0.9759	0.4841	2368608.0000
32	0.1993	0.9566	0.9258	0.9442	0.9344	1.7888	0.9851	0.9823	0.9896	0.9858	0.5145	1068317.7500
64	0.2272	0.9664	0.9666	0.9301	0.9456	1.5193	0.9902	0.9881	0.9948	0.9913	0.3749	556330.9375
Mean	0.1994	0.9451	0.9201	0.9175	0.9179	1.4388	0.9775	0.9735	0.9822	0.9777	0.4511	2185503.6719

Supplementary Note 4.2 Extended Results

The RMSE of each regression model across all window lengths are given in Fig. 7. In contrast to the novelty detector network, each expert regression model is able to reconstruct each intention class with good results across all time-windows. However, in this case we need that each intention class has enough richness such that the regression network can predict all possible scenarios. The results are summarized in Supplementary Table 7.

Fig. 8 reports some extended results of the regression bounds in a future time of 15 and 30 seconds using a window length of 8. Here, the results are promising, which can be enhanced by incorporating more data and variability. One advantage of the DMoE is that is flexible, that is, the expert model can be modified with novel architectures that possess better representation capabilities. This will clearly improve the final results of the DMoE and the predicted bounding boxes.

Supplementary Note 5 Physics Informed Reservoir Computing Derivation

Classical reservoir computing schemes [11] can suffer of poor representation capabilities due to the random initialization of the encoder weights associated to the input weights $\boldsymbol{W}_{in} \in \mathbb{R}^{n \times r}$ and reservoir weights $\boldsymbol{\mathcal{A}} \in \mathbb{R}^{r \times r}$. In this research, we aim to alleviate this robustness problem by incorporating physics informed feedback [12] to enhance the richness and heterogeneity of the reservoir high-dimensional representation.

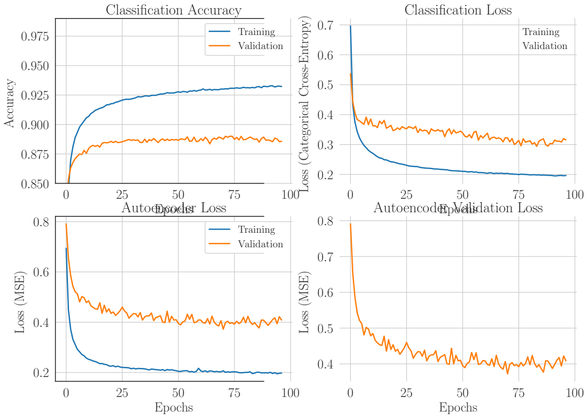

(a) Window length 8

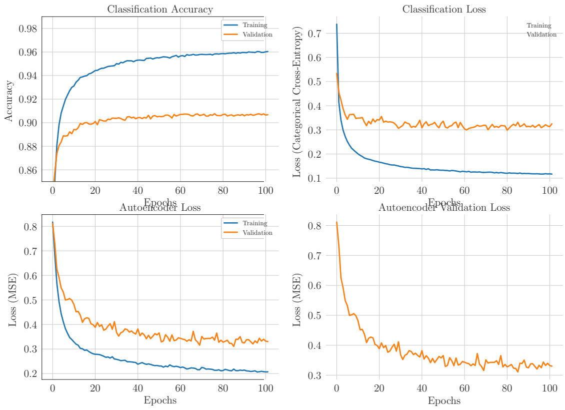

(b) Window length 16

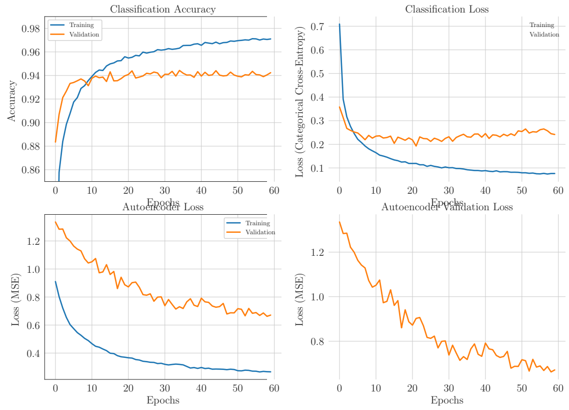

(c) Window length 32

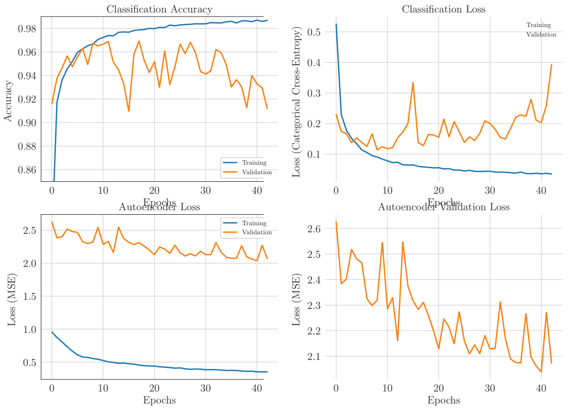

(d) Window length 64

Supplementary Figure 5: **Results of the Hybrid Classifier.** Testing results are plotted in blue solid lines and validation results are plotted in orange solid lines. Accuracy and loss results across different window lengths

Supplementary Note 5.1 General description

The general scheme of the proposed physics informed reservoir computing is given in Fig. 9. The diagram is composed of the standard reservoir computing scheme using a linear readout/decoder [13]. Here, we introduce an adaptive feedback loop given by the prediction error $\boldsymbol{e}$ between the estimation $\widehat{\boldsymbol{y}}$ and the real value $\boldsymbol{y}$ of the predictions, which allows to construct a non-linear affine system [14]. A feedback-linearization controller is then applied to the dynamics to achieve a stable performance. This control input is used to construct an adaptive reservoir weight matrix which is further applied to the reservoir computing network to enhance the robustness and high-dimensional representation capabilities.

Assume that the drone's trajectories can be exactly approximated by the following reservoir computing network [15]

$$\dot{\boldsymbol{y}} = \boldsymbol{W}_{out}\sigma(\boldsymbol{\mathcal{A}}^*\boldsymbol{r}^* + \boldsymbol{W}_{in}\boldsymbol{x}) + \boldsymbol{\varepsilon}, \tag{1}$$

where $\boldsymbol{\mathcal{A}}^* \in \mathbb{R}^{r\times r}$ defines the optimal reservoir weights matrix, $\boldsymbol{r}^* \in \mathbb{R}^r$ stands to the optimal reservoir states using r units, and $\boldsymbol{\varepsilon} \in \mathbb{R}^n$ is a bounded approximation error that can be decreased as the number of units in the reservoir layer increases. In addition, assume that the optimal reservoir weights can be written as $\boldsymbol{\mathcal{A}}^* = \boldsymbol{\mathcal{A}} + \boldsymbol{\mathcal{B}}^*$ for some unknown matrix $\boldsymbol{\mathcal{B}}^* \in \mathbb{R}^{r\times r}$. Then, the reservoir computing dynamics is slightly modified to

$$\dot{\widehat{\boldsymbol{y}}} = \boldsymbol{W}_{out}\sigma((\boldsymbol{\mathcal{A}} + \boldsymbol{\mathcal{B}})\boldsymbol{r} + \boldsymbol{W}_{in}\boldsymbol{x}), \tag{2}$$

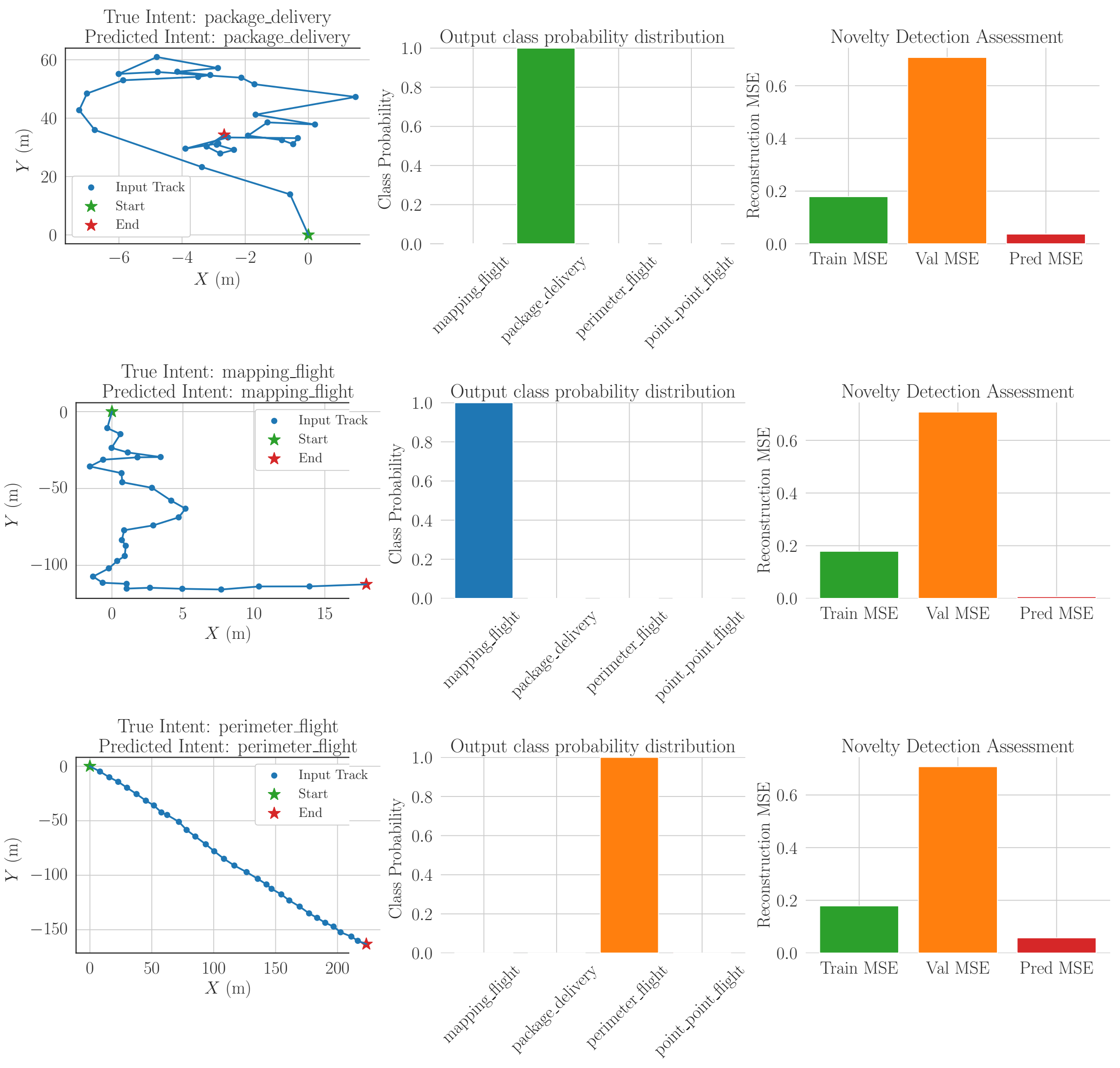

Supplementary Figure 6: **Trajectory Intention Classification extended results**. Trajectories are plotted with a blue doted line, the start point with a green star and the end point with a red star. The first and second rows show the classification and novelty detection assessment under the testing dataset using bar plots. The third row shows the classification and novelty detection assessment under unseen trajectories. The mean squared reconstruction error gives an indicator of potential malicious behaviour.

where $\boldsymbol{\mathcal{B}} \in \mathbb{R}^{r \times r}$ is a matrix that will be constructed from the physics informed model. Define the prediction error between the reservoir computing scheme and the input trajectories as $\boldsymbol{e} = \widehat{\boldsymbol{y}} - \boldsymbol{y} \in \mathbb{R}^n$. Then, the error dynamics is given by

$$\dot{e} = \boldsymbol{W}_{out}[\sigma(\boldsymbol{W}_{in}\boldsymbol{x} + (\boldsymbol{\mathcal{A}} + \boldsymbol{\mathcal{B}})\boldsymbol{r}) - \sigma(\boldsymbol{W}_{in}\boldsymbol{x} + \boldsymbol{\mathcal{A}}^*\boldsymbol{r}^*)] - \varepsilon. \tag{3}$$

Supplementary Note 5.2 Physics informed model analysis

Taylor series expansion is used in the reservoir dynamics around the vector $\boldsymbol{z}_0 := (\boldsymbol{\mathcal{A}} + \boldsymbol{\mathcal{B}})\boldsymbol{r} + \boldsymbol{W}_{in}\boldsymbol{x}$ as

$$\sigma(\boldsymbol{\mathcal{A}}^*\boldsymbol{r}^* + \boldsymbol{W}_{in}\boldsymbol{x}) \equiv \sigma(\boldsymbol{z}_0) + \boldsymbol{D}_\sigma(\boldsymbol{z}_0)(\boldsymbol{z} - \boldsymbol{z}_0) + \boldsymbol{\varepsilon}_\sigma \tag{4}$$

Supplementary Table 6: **Hyperparameters of the Trajectory intention regression**.

Parameter	DL models								
	Multi-Input LSTM		Multi-Input CNN		Multi-Input CBLSTMA			DMoE	
	Dense	RNN	Dense	CNN	Dense	RNN	CNN	Dense	CNN
Activations	ReLU	Tanh	ReLU	ReLU	ReLU	Tanh	ReLU	ReLU	ReLU
Hidden units	16	16	64	32	64	16	32	64	32
Units concat	64		64		64			64	
Dropout	0.4	0.4	-	0.4	0.4	0.4	-	0.4	-
Epochs	75		150		75			150	
Batch size	256		128		256			128	
Optimiser	Adam		Adam		Adam			Adam	
Learning rate	1e-4		5e-4		1e-3			5e-4	
Loss	Huber		Huber		Huber			Huber	

Supplementary Table 7: **Extended Trajectory intention regression results**. Best results per time window are in bold.

Model	Input Window Length (s)	Validation			Test		
		RMSE	MAE	R^2	RMSE	MAE	R^2
Multiple Linear Regressor	8	160.1633	78.2503	-0.3420	132.5540	77.3753	-0.5326
Multi-Input BLSTM		110.1799	41.7504	0.3842	85.4451	40.2597	0.5218
Multi-Input CNN		103.5907	39.0094	0.4657	85.3480	39.3726	0.3619
Multi-Input CBLSTMA		111.5730	41.5846	0.3653	87.3944	40.8109	0.2604
DMoE		**101.3812**	**31.3710**	**0.4863**	**68.6532**	**28.1353**	**0.7056**
Multiple Linear Regressor	16	153.6455	77.1540	-0.3620	132.7385	77.6763	-0.5332
Multi-Input BLSTM		112.2328	42.1724	0.2797	86.8703	39.9684	0.6193
Multi-Input CNN		101.3007	39.2357	0.4251	86.9849	40.5390	0.2960
Multi-Input CBLSTMA		106.4226	40.2257	0.3550	91.1821	41.0788	0.0145
DMoE		**93.7063**	**29.3688**	**0.5005**	**66.3241**	**27.1486**	**0.7996**
Multiple Linear Regressor	32	141.9160	75.2715	-0.4356	133.8516	78.9101	-0.5807
Multi-Input LSTM		99.8031	38.2608	0.2939	96.5178	42.0042	0.4052
Multi-Input CNN		83.8012	35.1835	0.5015	82.2166	38.4593	0.3680
Multi-Input CLSTMA		103.1382	40.0585	0.2450	88.9015	41.3739	0.1340
DMoE		**82.1361**	**25.7226**	**0.5235**	**65.8922**	**26.4180**	**0.8286**
Multiple Linear Regressor	64	118.1680	70.8340	-0.8352	150.1881	84.2064	-1.3223
Multi-Input BLSTM		74.7022	37.1499	0.2205	113.5891	50.7256	0.2842
Multi-Input CNN		69.8620	35.8270	0.3235	86.8546	43.0635	0.2564
Multi-Input CBLSTMA		65.0951	33.9712	0.4254	98.7680	45.2943	0.3227
DMoE		**59.2363**	**22.5724**	**0.5318**	**80.1402**	**30.3676**	**0.6589**

where $\boldsymbol{D}_\sigma(\boldsymbol{z}_0) = \frac{\partial\sigma(\boldsymbol{z})}{\partial \boldsymbol{z}}\Big|_{\boldsymbol{z}=\boldsymbol{z}_0} \in \mathbb{R}^{r\times r}$ and $\boldsymbol{\varepsilon}_\sigma \in \mathbb{R}^r$ is a second order approximation error. Then, the error dynamics (3) is equivalently written as

$$\begin{aligned}\dot{\boldsymbol{e}} =& \boldsymbol{W}_{out}\left[\boldsymbol{D}_\sigma(\boldsymbol{z}_0)((\boldsymbol{\mathcal{A}}+\boldsymbol{\mathcal{B}}^*)\boldsymbol{r}^* - (\boldsymbol{\mathcal{A}}+\boldsymbol{\mathcal{B}})\boldsymbol{r} + \boldsymbol{\varepsilon}_\sigma\right] - \boldsymbol{\varepsilon}\\ =& -\boldsymbol{W}_{out}\boldsymbol{D}_\sigma(\boldsymbol{z}_0)[\boldsymbol{\mathcal{A}}\widetilde{\boldsymbol{r}} + \boldsymbol{\mathcal{B}}\widetilde{\boldsymbol{r}}] + \bar{\boldsymbol{\varepsilon}},\end{aligned} \tag{5}$$

where $\bar{\boldsymbol{\varepsilon}} = -\boldsymbol{W}_{out}\boldsymbol{D}_\sigma(\boldsymbol{z}_0)[\widetilde{\boldsymbol{\mathcal{B}}}\boldsymbol{r}^* - \boldsymbol{\varepsilon}_\sigma] - \boldsymbol{\varepsilon} \in \mathbb{R}^n$, $\widetilde{\boldsymbol{\mathcal{B}}} = \boldsymbol{\mathcal{B}} - \boldsymbol{\mathcal{B}}^* \in \mathbb{R}^{r\times r}$ and $\widetilde{\boldsymbol{r}} = \boldsymbol{r} - \boldsymbol{r}^* \in \mathbb{R}^r$ stand for the error matrix and the error of the reservoir states. Here $\boldsymbol{r}^*$ can be computed with $\boldsymbol{r}^* = \boldsymbol{W}_{out}^\dagger(\boldsymbol{y} - \boldsymbol{w})$.

Define

$$\begin{aligned}\boldsymbol{f}(\boldsymbol{r},\boldsymbol{x}) =& \quad -\boldsymbol{W}_{out}\boldsymbol{D}_\sigma(\boldsymbol{z}_0)\boldsymbol{\mathcal{A}}\widetilde{\boldsymbol{r}} \in \mathbb{R}^n,\\ \boldsymbol{g}(\boldsymbol{r},\boldsymbol{x}) =& \quad -\boldsymbol{W}_{out}\boldsymbol{D}_\sigma(\boldsymbol{z}_0) \otimes \widetilde{\boldsymbol{r}}^\top \in \mathbb{R}^{n\times r^2}\\ \boldsymbol{u} =& \quad \mathrm{vec}(\boldsymbol{B}_1).\end{aligned} \tag{6}$$

where $\boldsymbol{B}_1 \in \mathbb{R}^{r\times r}$ is an auxiliary matrix used to stabilize the error dynamics. For instance, we can assume that $\boldsymbol{B}_1$ is equivalent to $\boldsymbol{\mathcal{B}}$. The error dynamics (5) can be equivalently written as

$$\dot{\boldsymbol{e}} = \boldsymbol{f}(\boldsymbol{x},\boldsymbol{r}) + \boldsymbol{g}(\boldsymbol{x},\boldsymbol{r})\boldsymbol{u} + \bar{\boldsymbol{\varepsilon}}. \tag{7}$$

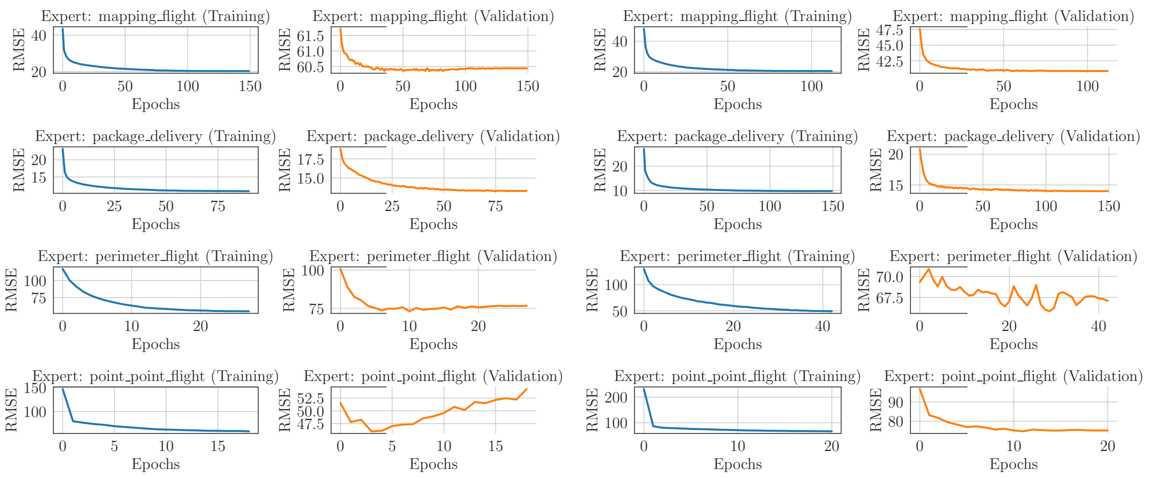

(a) Window length 8 (b) Window length 16

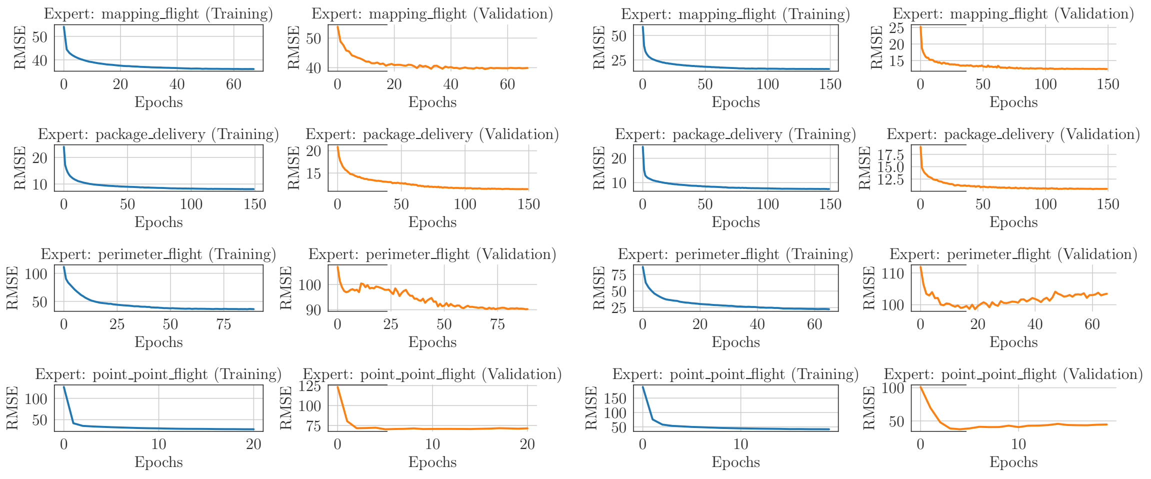

(c) Window length 32 (d) Window length 64

Supplementary Figure 7: **Trajectory Intention Regression results**. Training results are reported in blue solid line, and validation results are reported in orange solid line. RMSE of each expert regression model

Then, the control input $\boldsymbol{u}$ can be computed as

$$\boldsymbol{u} = -\boldsymbol{g}^{\dagger}(\boldsymbol{x}, \boldsymbol{r}) \left(\boldsymbol{f}(\boldsymbol{x}, \boldsymbol{r}) + \boldsymbol{\mathcal{K}} \boldsymbol{e} \right), \tag{8}$$

where $\boldsymbol{\mathcal{K}} \in \mathbb{R}^{n \times n}$ is a diagonal matrix gain which is tuned small enough to avoid noise excitation. The error dynamics (7) in closed-loop with the control input (8) is

$$\dot{\boldsymbol{e}} = -\boldsymbol{\mathcal{K}} \boldsymbol{e} + \bar{\boldsymbol{\varepsilon}}. \tag{9}$$

The next theorem establishes the uniform ultimately boundedness (UUB) [16] of the prediction error trajectories under the proposed PIRC.

Theorem 1. The prediction error trajectories (7) under the control input (8) exhibit practical stability and converge to a bounded set S_μ of radius $\mu = \frac{\|\bar{\boldsymbol{\varepsilon}}\|_2}{\lambda_{\min}(\boldsymbol{\mathcal{K}})}$ as $t \to \infty$ and hence, the prediction error trajectories $\boldsymbol{e}$ are UUB.

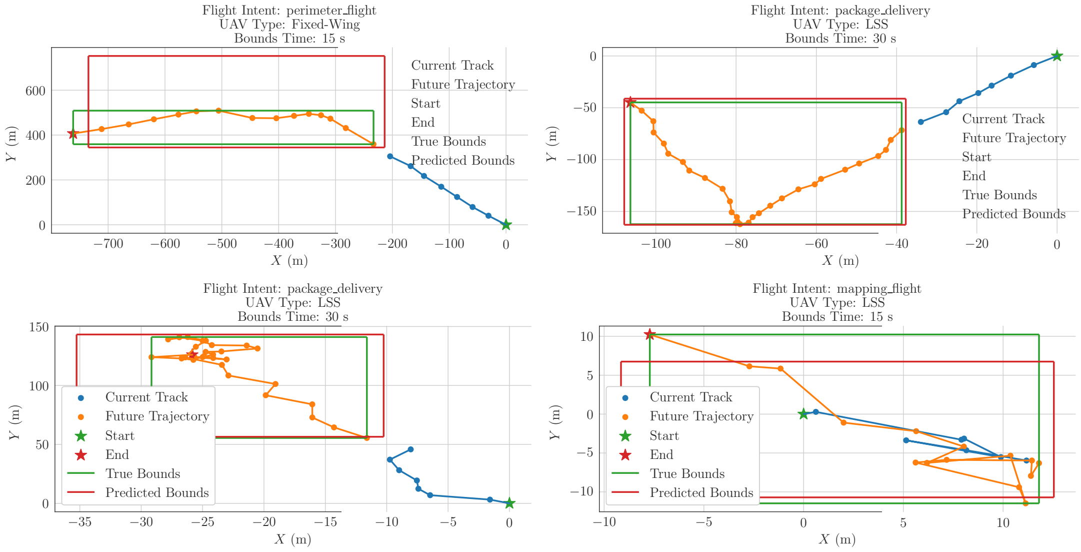

Supplementary Figure 8: **Trajectory Intention Regression extended results.** Each figure represents one of the four trajectory intention classes. The blue dotted line is the current trajectory track, the orange dotted line is the future trajectory, the start point is given by a green star, and the end point is given by a red star. The green solid line bounding box corresponds to the true future airspace and the red dotted line bounding box corresponds to the predicted future airspace that will occupy the drone.

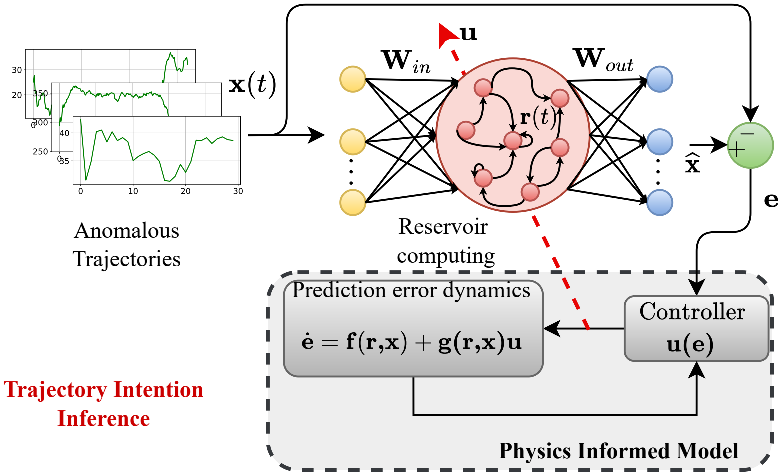

Supplementary Figure 9: **Physics Informed Reservoir Computing Scheme**

Proof. The following Lyapunov function is considered

$$V = \frac{1}{2}\boldsymbol{e}^\top \boldsymbol{e}, \tag{10}$$

The time-derivative of (10) along the prediction error trajectories (9) is

$$\begin{aligned}\dot{V} &= -\boldsymbol{e}^\top \boldsymbol{\mathcal{K}} \boldsymbol{e} + \boldsymbol{e}^\top \bar{\boldsymbol{\varepsilon}} \\ &\leq -\lambda_{\min}(\boldsymbol{\mathcal{K}})\|\boldsymbol{e}\|_2^2 + \|\bar{\boldsymbol{\varepsilon}}\|_2\|\boldsymbol{e}\|_2 \\ &= -\lambda_{\min}(\boldsymbol{\mathcal{K}})\|\boldsymbol{e}\|_2\Big(\|\boldsymbol{e}\|_2 - \frac{\|\bar{\boldsymbol{\varepsilon}}\|_2}{\lambda_{\min}(\boldsymbol{\mathcal{K}})}\Big).\end{aligned} \tag{11}$$

$\dot{V}$ is negative definite if

$$\|\boldsymbol{e}\|_2 > \frac{\|\bar{\boldsymbol{\varepsilon}}\|_2}{\lambda_{\min}(\boldsymbol{\mathcal{K}})} \equiv \mu. \tag{12}$$

Therefore, there exists a large enough $\boldsymbol{\mathcal{K}}$ that ensures that the error trajectories (9) converge into a bounded set S_μ of radius $\mu = \frac{\|\bar{\boldsymbol{\varepsilon}}\|_2}{\lambda_{\min}(\boldsymbol{\mathcal{K}})}$, i.e., as $t \to \infty$ and hence, the prediction error trajectories of $\boldsymbol{e}$ are UUB. This completed the proof. □

The stabilizing control $\boldsymbol{u}$ is transformed into a matrix of dimension $r \times r$ using

$$\boldsymbol{B}_1 = \mathrm{mat}(\boldsymbol{u}). \tag{13}$$

It is clear that, by construction, $\boldsymbol{B}_1$ is a matrix that can compromise the stability of the initial weight matrix $\boldsymbol{\mathcal{A}}$. To solve this issue, we construct a negative semi-definite matrix from $\boldsymbol{B}_1$ as

$$\boldsymbol{\mathcal{B}} = -\frac{1}{2}\boldsymbol{B}_1^\top \boldsymbol{B}_1. \tag{14}$$

From experimentation, we observe that this matrix can cause that the eigenvalues of the reservoir weights to be close to the unstable real margin causing divergence of the reservoir states. To overcome this issue, we add a small diagonal matrix to ensure negative definiteness and eigenvalues that are not close to zero. Therefore, the adaptive reservoir weights are

$$\boldsymbol{\mathcal{B}} = -\frac{1}{2}(\boldsymbol{B}_1^\top \boldsymbol{B}_1 + \epsilon \boldsymbol{I}_r), \tag{15}$$

where $\epsilon > 0$ is a small positive scalar. In this research, this scalar is determined in accordance with the future-time window f_t of prediction. Here, we propose $\epsilon = 0.0001 f_t$.

Supplementary Note 5.3 Hyperparameters

The hyperparameters used for the trajectory prediction algorithms are summarized in Supplementary Table 8.

Supplementary Table 8: **Hyperparameters of the Reservoir Computing Predictors**

Model	Hyperparameters		
	Reservoir Units	Kernel	Hidden Units
RC Linear	30	-	-
RC SVM		RBF	-
RC MLP		-	[10,30,10]
PIRC		-	-

Supplementary Note 5.4 Extended results

Fig. 10 shows additional prediction results of the linear RC and PIRC under different mission profiles. The prediction window is of 100 steps. The results show that the algorithm works well using the predefined number of reservoir units. The results can be enhanced by tuning this hyperparameter and the scalar ϵ of the PIRC.

Table 9 shows the MSE results of one real-world mission profile across all time-windows and different reservoir computing configurations. Here, the results clearly shows that the proposed PIRC can improve stability and precision in the predictions. For large time-windows, the RC with linear decoder tends to diverge, however the physics-informed loop helps to regularize the learning manifold.

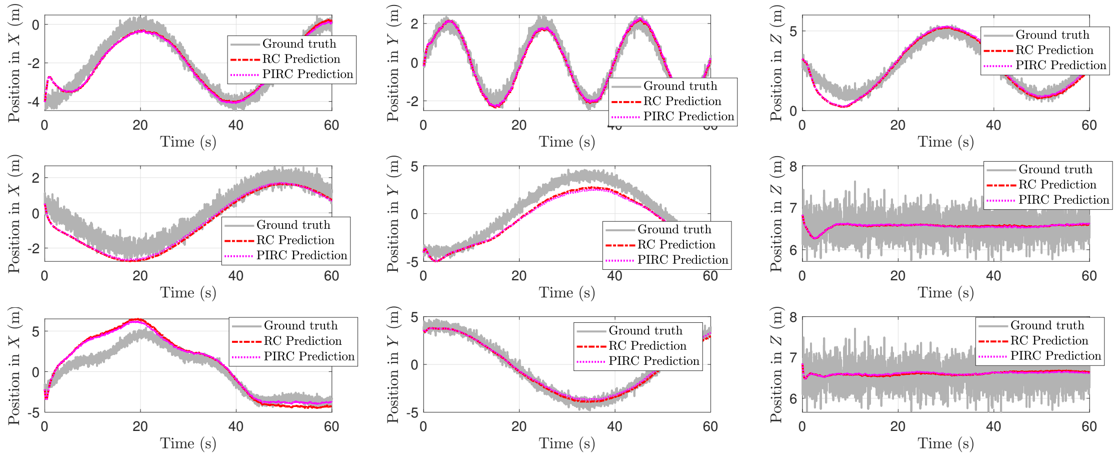

Supplementary Figure 10: **Trajectory prediction additional results**. Ground truth is plotted with a gray solid line, RC prediction results in red dashed line, and PIRC in pink dashed line. Results for a prediction time window of 100 steps. Each column represents the positions in the X, Y, Z directions.

Supplementary Table 9: **Mean Squared error results across different future time windows**.

Prediction Window (steps)	Mean Squared Error (MSE)							
	RC Linear [17]		RC SVM [13]		RC MLP [11]		PIRC (ours)	
	Train	Test	Train	Test	Train	Test	Train	Test
1	0.0168	0.0218	**0.0162**	1.3664	0.0165	0.2114	0.0170	**0.0214**
10	0.0169	0.0236	**0.0161**	1.3644	0.0166	0.0756	0.0170	**0.0235**
100	0.0173	**0.0459**	**0.0161**	1.3614	0.0168	0.1566	0.0200	0.0460
1,000	0.0168	0.0285	0.0163	0.4841	**0.0161**	0.2195	0.0244	**0.0262**
Average	0.0169	0.0299	**0.0162**	1.1441	0.0165	0.1658	0.0196	**0.0293**

Best results for training are in bold and best results for testing are in bold and underlined.

Supplementary Note 6 Off-policy Model-based Reward-Shaping Inverse Reinforcement Learning

One of the major hypothesis in this research is that the controller contains information about the mission profile that exhibits the drone and its main intent. This information is hidden within the control structure in the form of a scalar reward function or reward function. Here, we aim to uncover this reward function to obtain a proxy indicator of misbehaviour.

Supplementary Note 6.1 DMDc additional results

Dynamic Mode Decomposition with Control (DMDc) [18] is used to obtain a linear system that preserves the nonlinear properties of the drone dynamics and facilitates the incorporation of the control signal as a regularisation term of the learning manifold. To this end, we use as training data the trajectories that exhibit more richness to ensure a consistent linear model. As an example, the

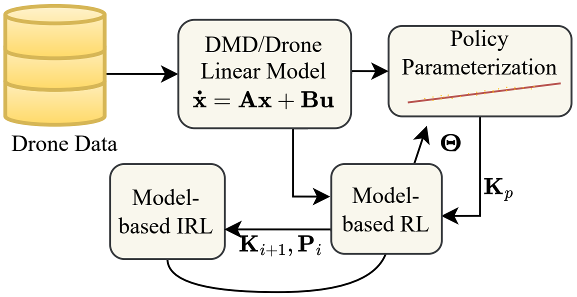

Supplementary Figure 11: **Reward intention inference scheme.**

following linear matrices of a particular mission profile are obtained

$$\boldsymbol{A}_D = \begin{bmatrix} 0.9897 & 0.0012 & -0.0004 & 0.0090 & -0.0038 & 0.0007 \\ 0.0003 & 0.9874 & 0.0021 & 0.0003 & 0.0083 & 0.0003 \\ -0.0006 & 0.0032 & 0.9956 & -0.0024 & -0.0008 & 0.0101 \\ 0.0017 & 0.0038 & 0.0020 & 0.8583 & 0.0126 & 0.0007 \\ -0.0015 & -0.0010 & -0.0004 & 0.0156 & 0.9110 & 0.0057 \\ 0.0006 & 0.0028 & -0.0038 & -0.0021 & 0.0040 & 0.9902 \end{bmatrix},$$

$$\boldsymbol{B}_D = \begin{bmatrix} -0.1112 & -0.1090 & -0.0193 & -0.0001 \\ 0.2629 & 0.0223 & -0.0923 & 0.0001 \\ -0.1387 & -0.0827 & -0.1097 & -0.0002 \\ -0.4133 & -0.0658 & -0.0131 & 0.0010 \\ -0.0059 & -0.0536 & -0.0880 & -0.0002 \\ -0.1198 & 0.1249 & -0.2082 & 0.0002 \end{bmatrix}.$$

With these matrices we design a discrete linear quadratic regulator (LQR) to ensure trajectory tracking. One important aspect for the control design is that the Euler angles (mainly roll and pitch movements) must be small to verify a linear performance, i.e., the Euler angles remain within $[-\frac{\pi}{6}, \frac{\pi}{6}]$, which is consistent with the collected data. For this particular example, we set $\boldsymbol{Q} = \text{diag}\{1, 1, 1, 0.01, 0.01, 0.01\}$ and $\boldsymbol{R} = 0.01\boldsymbol{I}_4$. The obtained control gain is

$$\boldsymbol{K} = \begin{bmatrix} 2.3711 & 3.1683 & -2.8436 & 0.0343 & 0.0191 & -0.0426 \\ -10.8777 & -2.6365 & 3.4413 & -0.1276 & 0.0194 & 0.0701 \\ 4.4412 & -2.0712 & -7.3452 & 0.0699 & -0.0330 & -0.1034 \\ 0.0462 & 0.0138 & -0.0480 & 0.0071 & 0.0005 & 0.0099 \end{bmatrix}.$$

Fig. 12 shows additional results of the linear model predictor using the DMDc method and the proposed LQR controller. Here, the results show that the obtained linear model under the proposed discrete LQR is able to capture the dynamics properties [19] of the drone nonlinear dynamics and can follow the desired reference accurately. Here it is important to mention that the richness of the training data is crucial to ensure that the linear model predictor has the enough representation capabilities of the drone dynamics.

Supplementary Note 6.2 Direct Optimal Control

First, lets describe the optimal control problem [20] where the reward function appears naturally. The drone is modelled as a linear system of the form

$$\dot{\boldsymbol{x}} = \boldsymbol{A}\boldsymbol{x} + \boldsymbol{B}\boldsymbol{u}, \quad \boldsymbol{x}(t_0) = \boldsymbol{x}_0, \tag{16}$$

where $\boldsymbol{A} \in \mathbb{R}^{n \times n}$ and $\boldsymbol{B} \in \mathbb{R}^{n \times m}$ define the dynamics of the drone. It is assumed that the drone is controlled by an optimal controller which is designed to minimize the following quadratic cost

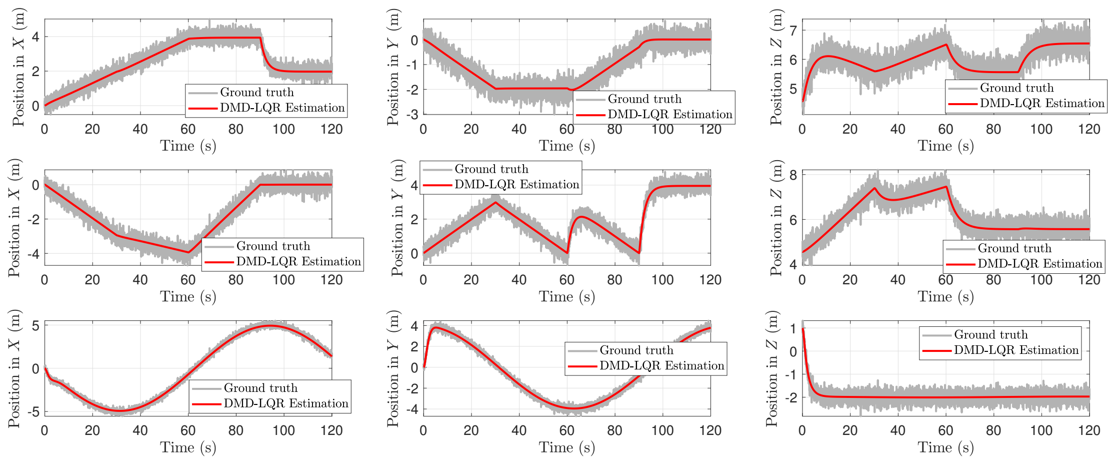

Supplementary Figure 12: **Linear model predictor**. Ground truth is plotted in gray solid line and DMD-LQR estimation in a red solid line. DMDc results across different mission profiles. Each column represents the positions in the X, Y, Z directions.

index [21]

$$J = \int_t^\infty \left((\boldsymbol{x}^d - \boldsymbol{x})^\top \boldsymbol{Q} (\boldsymbol{x}^d - \boldsymbol{x}) + \boldsymbol{u}^\top \boldsymbol{R} \boldsymbol{u} \right) d\tau, \tag{17}$$

where $\boldsymbol{Q} = \boldsymbol{Q}^\top \geq 0 \in \mathbb{R}^{n \times n}$ and $\boldsymbol{R} = \boldsymbol{R}^\top > 0$ are positive semi-definite and definite weight matrices, and $\boldsymbol{x}^d \in \mathbb{R}^n$ is a desired reference. The reward function $\xi \in \mathbb{R}$ is the term inside the integral of the above cost, i.e.,

$$\xi(\boldsymbol{x}, \boldsymbol{x}^d, \boldsymbol{u}) = (\boldsymbol{x}^d - \boldsymbol{x})^\top \boldsymbol{Q} (\boldsymbol{x}^d - \boldsymbol{x}) + \boldsymbol{u}^\top \boldsymbol{R} \boldsymbol{u}. \tag{18}$$

The optimal control that minimizes the above cost is given by

$$\boldsymbol{u} = \boldsymbol{R}^{-1} \boldsymbol{B}^\top \boldsymbol{P} (\boldsymbol{x}^d - \boldsymbol{x}) = \boldsymbol{K} (\boldsymbol{x}^d - \boldsymbol{x}), \tag{19}$$

where $\boldsymbol{K} = \boldsymbol{R}^{-1} \boldsymbol{B}^\top \boldsymbol{P} \in \mathbb{R}^{m \times n}$ is a stabilizing gain and $\boldsymbol{P} = \boldsymbol{P}^\top > 0 \in \mathbb{R}^{n \times n}$ is a kernel matrix that verifies the following Algebraic Riccati equation (ARE)

$$\boldsymbol{A}^\top \boldsymbol{P} + \boldsymbol{P} \boldsymbol{A} + \boldsymbol{Q} - \boldsymbol{P} \boldsymbol{B} \boldsymbol{R}^{-1} \boldsymbol{B}^\top \boldsymbol{P} = \boldsymbol{0}_{n \times n}. \tag{20}$$

Supplementary Note 6.3 Policy Parameterization

Assume that we have matrices constructed from the collection of ι measurements of the drone's states, control inputs and reward function of a random mission profile [18], i.e.,

$$\begin{aligned} \boldsymbol{X} &= \begin{bmatrix} | & | & & | \\ \boldsymbol{x}_1 & \boldsymbol{x}_2 & \cdots & \boldsymbol{x}_{\iota-1} \\ | & | & & | \end{bmatrix}, \quad \boldsymbol{\Upsilon} = \begin{bmatrix} | & | & & | \\ \boldsymbol{u}_1 & \boldsymbol{u}_2 & \cdots & \boldsymbol{u}_{\iota-1} \\ | & | & & | \end{bmatrix}, \\ \boldsymbol{X}^d &= \begin{bmatrix} | & | & & | \\ \boldsymbol{x}_1^d & \boldsymbol{x}_2^d & \cdots & \boldsymbol{x}_{\iota-1}^d \\ | & | & & | \end{bmatrix}, \quad \boldsymbol{\Xi} = \begin{bmatrix} \xi_1 & \xi_2 & \cdots & \xi_{\iota-1} \end{bmatrix}. \end{aligned} \tag{21}$$

The control gain is linearly parameterized as

$$\boldsymbol{\Upsilon} = \boldsymbol{K}_p (\boldsymbol{X}^d - \boldsymbol{X}), \tag{22}$$

then the control gain is obtained using a batch-least squares rule given by

$$\boldsymbol{K}_p = \boldsymbol{\Upsilon} (\boldsymbol{X}^d - \boldsymbol{X})^\dagger, \tag{23}$$

where $\boldsymbol{K}_p$ is an estimate of $\boldsymbol{K}$. If the measurements are free of noise then $\boldsymbol{K}_p \equiv \boldsymbol{K}$.

Supplementary Note 6.4 Reward-Shaping Inverse Reinforcement Learning

Define $\boldsymbol{K}_p \in \mathbb{R}^{m\times n}$, $\boldsymbol{P}_p \in \mathbb{R}^{n\times n}$, $\boldsymbol{Q}_p \in \mathbb{R}^{n\times n}$ and $\boldsymbol{R}_p \in \mathbb{R}^{m\times m}$ as the real gain and weight matrices of the optimal control problem. We aim to infer the weight matrices of the reward function from the knowledge of matrices $\boldsymbol{A}$ and $\boldsymbol{B}$ and matrices (21). The proposed model-based reward-shaping inverse reinforcement learning algorithm is given in Algorithm 1

Algorithm 1 Off-line Model-based Inverse Reinforcement Learning

1: Collect measurements of $\boldsymbol{X} \in \mathbb{R}^{n\times\iota}$, $\boldsymbol{X}^d \in \mathbb{R}^{n\times\iota}$, and $\boldsymbol{\Xi} \in \mathbb{R}^{1\times\iota}$. Select $\boldsymbol{Q}_0 = \boldsymbol{Q}_0^\top > 0$ and $\boldsymbol{R}_0 = \boldsymbol{R}_0^\top > 0$, and a stabilizing gain $\boldsymbol{K}_{\boldsymbol{0}}$. Set $i = 0$ and a small threshold ε_k.

2: **Policy Evaluation.** Compute $\boldsymbol{P}_i$

$$\boldsymbol{0}_{n\times n} = (\boldsymbol{A} - \boldsymbol{B}\boldsymbol{K}_i)^\top \boldsymbol{P}_i + \boldsymbol{P}_i(\boldsymbol{A} - \boldsymbol{B}\boldsymbol{K}_i) + \boldsymbol{K}_i^\top \boldsymbol{R}_i \boldsymbol{K}_i - (\boldsymbol{K}_i - \boldsymbol{K}_p)^\top \boldsymbol{R}_i (\boldsymbol{K}_i - \boldsymbol{K}_p) + \boldsymbol{Q}_i. \tag{24}$$

3: **Policy Improvement.** Compute $\boldsymbol{K}_{i+1}$

$$\boldsymbol{K}_{i+1} = \boldsymbol{R}_i^{-1}\boldsymbol{B}^\top \boldsymbol{P}_i. \tag{25}$$

4: **Weights Improvement**. Compute $\boldsymbol{\Theta} = [\mathrm{vec}(\boldsymbol{Q}_{i+1})^\top, \mathrm{vec}(\boldsymbol{R}_{i+1})^\top]^\top$

$$\begin{bmatrix} \boldsymbol{I}_{n^2} & -(\boldsymbol{K}_{i+1} \otimes \boldsymbol{K}_{i+1})^\top \\ \boldsymbol{I}_{n^2} & (\boldsymbol{K}_p \otimes \boldsymbol{K}_p)^\top \end{bmatrix} \boldsymbol{\Theta} = \begin{bmatrix} -\mathrm{vec}(\boldsymbol{A}^\top \boldsymbol{P}_i + \boldsymbol{P}_i \boldsymbol{A}) \\ [(\boldsymbol{X}^{\boldsymbol{d}} - \boldsymbol{X})^\top \otimes (\boldsymbol{X}^d - \boldsymbol{X})^\top]^\dagger \boldsymbol{\Xi}^\top \end{bmatrix}. \tag{26}$$

5: **Stop the algorithm** if $\|\boldsymbol{K}_{i+1} - \boldsymbol{K}_i\| \leq \varepsilon_k$, otherwise set $i = i + 1$ and return to Step 2.

Supplementary Note 6.4.1 Reinforcement Learning Update Analysis

Let rewrite the ARE (20) using a stabilizing control gain $\boldsymbol{K}_i$ as

$$\begin{aligned} \boldsymbol{Q}_p &= -(\boldsymbol{A} - \boldsymbol{B}\boldsymbol{K}_i)^\top \boldsymbol{P}_p - \boldsymbol{P}_p(\boldsymbol{A} - \boldsymbol{B}\boldsymbol{K}_i) + \boldsymbol{K}_p^\top \boldsymbol{R}_p \boldsymbol{K}_p - \boldsymbol{K}_i^\top \boldsymbol{R}_p \boldsymbol{K}_p - \boldsymbol{K}_p^\top \boldsymbol{R}_p \boldsymbol{K}_i, \\ \boldsymbol{Q}_p &= -(\boldsymbol{A} - \boldsymbol{B}\boldsymbol{K}_i)^\top \boldsymbol{P}_p - \boldsymbol{P}_p(\boldsymbol{A} - \boldsymbol{B}\boldsymbol{K}_i) + (\boldsymbol{K}_i - \boldsymbol{K}_p)^\top \boldsymbol{R}_p (\boldsymbol{K}_i - \boldsymbol{K}_p) - \boldsymbol{K}_i^\top \boldsymbol{R}_p \boldsymbol{K}_i. \end{aligned} \tag{27}$$

Without loss of generality, the ARE (27) can be written in terms of the following iterative matrices $\boldsymbol{P}_i$, $\boldsymbol{Q}_i$, and $\boldsymbol{R}_i$ as

$$\boldsymbol{Q}_i = -(\boldsymbol{A} - \boldsymbol{B}\boldsymbol{K}_i)^\top \boldsymbol{P}_i - \boldsymbol{P}_i(\boldsymbol{A} - \boldsymbol{B}\boldsymbol{K}_i) + (\boldsymbol{K}_i - \boldsymbol{K}_p)^\top \boldsymbol{R}_i (\boldsymbol{K}_i - \boldsymbol{K}_p) - \boldsymbol{K}_i^\top \boldsymbol{R}_i \boldsymbol{K}_i. \tag{28}$$

The ARE (28) is equivalent to (24) in Algorithm 1. The difference between the ARE (27) and the (28) gives

$$\begin{aligned} \boldsymbol{Q}_p =& \boldsymbol{Q}_i + (\boldsymbol{A} - \boldsymbol{B}\boldsymbol{K}_i)^\top (\boldsymbol{P}_i - \boldsymbol{P}_p) + (\boldsymbol{P}_i - \boldsymbol{P}_p)(\boldsymbol{A} - \boldsymbol{B}\boldsymbol{K}_i) - (\boldsymbol{K}_i - \boldsymbol{K}_p)^\top (\boldsymbol{R}_i - \boldsymbol{R}_p)(\boldsymbol{K}_i - \boldsymbol{K}_p) \\ &+ \boldsymbol{K}_i^\top (\boldsymbol{R}_i - \boldsymbol{R}_p)\boldsymbol{K}_i. \end{aligned} \tag{29}$$

Notice that our goal is to show that

$$\boldsymbol{Q}_p \geq \boldsymbol{Q}_i - (\boldsymbol{K}_i - \boldsymbol{K}_p)^\top (\boldsymbol{R}_i - \boldsymbol{R}_p)(\boldsymbol{K}_i - \boldsymbol{K}_p) + \boldsymbol{K}_i^\top (\boldsymbol{R}_i - \boldsymbol{R}_p)\boldsymbol{K}_i. \tag{30}$$

By construction the term $(\boldsymbol{A} - \boldsymbol{B}\boldsymbol{K}_i)$ is Hurwitz such that

$$(\boldsymbol{A} - \boldsymbol{B}\boldsymbol{K}_i)^\top (\boldsymbol{P}_i - \boldsymbol{P}_p) + (\boldsymbol{P}_i - \boldsymbol{P}_p)(\boldsymbol{A} - \boldsymbol{B}\boldsymbol{K}_i) \leq \boldsymbol{0}_{n\times n}, \tag{31}$$

which is valid if and only if $\boldsymbol{P}_i \geq \boldsymbol{P}_p$ for all iterative matrix $\boldsymbol{P}_i$ such that (30) is satisfied. However, since $\boldsymbol{R}_i$ is not fixed, then multiple combinations between $\boldsymbol{Q}_i$ and $\boldsymbol{R}_i$ can ensure that $\boldsymbol{K}_i$ converges to

$\boldsymbol{K}_p$. Consider now the first row of the weights improvement (26) written in terms of iterative control gain $\boldsymbol{K}_i$ and the policy improvement (25)

$$\boldsymbol{Q}_{i+1} = -(\boldsymbol{A}-\boldsymbol{B}\boldsymbol{K}_i)^\top \boldsymbol{P}_i - \boldsymbol{P}_i(\boldsymbol{A}-\boldsymbol{B}\boldsymbol{K}_i) + \boldsymbol{K}_{i+1}^\top \boldsymbol{R}_{i+1}\boldsymbol{K}_{i+1} - \boldsymbol{K}_i^\top \boldsymbol{R}_i \boldsymbol{K}_{i+1} - \boldsymbol{K}_{i+1}^\top \boldsymbol{R}_i \boldsymbol{K}_i. \tag{32}$$

The difference between (32) and (28) gives

$$\boldsymbol{Q}_{i+1} = \boldsymbol{Q}_i + \boldsymbol{K}_{i+1}^\top(\boldsymbol{R}_{i+1}-\boldsymbol{R}_i)\boldsymbol{K}_{i+1} + (\boldsymbol{K}_{i+1}-\boldsymbol{K}_i)^\top \boldsymbol{R}_i(\boldsymbol{K}_{i+1}-\boldsymbol{K}_i) - (\boldsymbol{K}_i-\boldsymbol{K}_p)^\top \boldsymbol{R}_i(\boldsymbol{K}_i-\boldsymbol{K}_p). \tag{33}$$

From the above result it follows that $\boldsymbol{Q}_{i+1} \geq \boldsymbol{Q}_i$ such that given a small or inclusive zero initial matrix $\boldsymbol{Q}_0$ (because the pair $(\boldsymbol{0}_{n\times n}, \boldsymbol{A}-\boldsymbol{B}\boldsymbol{K}_0)$ is observable) it follows that $\boldsymbol{0}_{n\times n} = \boldsymbol{Q}_0 \leq \boldsymbol{Q}_1 \leq \cdots \leq \boldsymbol{Q}_i \leq \boldsymbol{Q}_{i+1} \leq \cdots \leq \boldsymbol{Q}_p$. Notice that as $\boldsymbol{K}_i$ approaches to $\boldsymbol{K}_p$ then $\boldsymbol{P}_i$ is unchanged in each iteration and hence $\boldsymbol{K}_{i+1}$ is equivalent to $\boldsymbol{K}_i$ such that $\boldsymbol{Q}_{i+1}$ converges to $\boldsymbol{Q}_{i+1} = \boldsymbol{Q}_i + \boldsymbol{K}_p^\top(\boldsymbol{R}_{i+1}-\boldsymbol{R}_i)\boldsymbol{K}_p$. This demonstrates that multiple weight matrices $\boldsymbol{Q}_i$ and $\boldsymbol{R}_i$ can achieve the same control gain $\boldsymbol{K}_i$. In this research, the reward values are used to avoid multiple solutions and ensure convergence to their real values. This is discussed in the IRL algorithm analysis.

Supplementary Note 6.4.2 Inverse Reinforcement Learning Update Analysis

Consider the first row of the weights improvement (26) written as

$$\boldsymbol{Q}_{i+1} - \boldsymbol{K}_{i+1}^\top \boldsymbol{R}_{i+1}\boldsymbol{K}_{i+1} = -\boldsymbol{A}^\top \boldsymbol{P}_i - \boldsymbol{P}_i\boldsymbol{A}. \tag{34}$$

Here, the terms in the right hand side of the equality have full rank, i.e., $\mathrm{rank}(\boldsymbol{A}^\top\boldsymbol{P}_i + \boldsymbol{P}_i\boldsymbol{A}) = n$. However, we aim to estimate the n^2 elements of the weight matrix $\boldsymbol{Q}_i$ and the m^2 elements of matrix $\boldsymbol{R}_i$, such that (34) is insufficient and, in consequence, a combination of weights is obtained without any constraint. In the worst case, the estimated matrices are negative definite and cannot provide an adequate indicator for potential misbehaviour.

To this end, we make use of the reward function values to incorporate ι equality constraints to the algorithm in order to ensure convergence to the exact real values. Here, we have the following

$$\begin{aligned}
\boldsymbol{\Xi} =& (\boldsymbol{X}^d-\boldsymbol{X})^\top(\boldsymbol{Q}_{i+1} + \boldsymbol{K}_p^\top \boldsymbol{R}_{i+1}\boldsymbol{K}_p)(\boldsymbol{X}^d-\boldsymbol{X}) \\
\boldsymbol{\Xi}^\top =& (\boldsymbol{X}^d-\boldsymbol{X})^\top \otimes (\boldsymbol{X}^d-\boldsymbol{X}^\top)\begin{bmatrix}\boldsymbol{I}_{n^2} & (\boldsymbol{K}_p\otimes\boldsymbol{K}_p)^\top\end{bmatrix}\boldsymbol{\Theta} \\
\begin{bmatrix}\boldsymbol{I}_{n^2} & (\boldsymbol{K}_p\otimes\boldsymbol{K}_p)^\top\end{bmatrix}\boldsymbol{\Theta} =& [(\boldsymbol{X}^d-\boldsymbol{X})^\top \otimes (\boldsymbol{X}^d-\boldsymbol{X})^\top]^\dagger \boldsymbol{\Xi}^\top,
\end{aligned} \tag{35}$$

where $\boldsymbol{\Theta} = [\mathrm{vec}(\boldsymbol{Q}_{i+1})^\top, \mathrm{vec}(\boldsymbol{R}_{i+1})^\top]^\top \in \mathbb{R}^{n^2+m^2}$. Notice that the matrix $\begin{bmatrix}\boldsymbol{I}_n^2, (\boldsymbol{K}_p\otimes\boldsymbol{K}_p)^\top\end{bmatrix} \in \mathbb{R}^{n^2\times(n^2+m^2)}$ such that it faces the same rank problem as (34). Therefore, we concatenate (35) and (34) to construct a matrix of rank n^2+m^2. First, (34) is rewritten as

$$\begin{bmatrix}\boldsymbol{I}_{n^2} & -(\boldsymbol{K}_{i+1}\otimes\boldsymbol{K}_{i+1})^\top\end{bmatrix}\boldsymbol{\Theta} = -\mathrm{vec}(\boldsymbol{A}^\top\boldsymbol{P}_i + \boldsymbol{P}_i\boldsymbol{A}). \tag{36}$$

Then concatenating (35) and (36) gives

$$\underbrace{\begin{bmatrix}\boldsymbol{I}_{n^2} & -(\boldsymbol{K}_{i+1}\otimes\boldsymbol{K}_{i+1})^\top \\ \boldsymbol{I}_{n^2} & (\boldsymbol{K}_p\otimes\boldsymbol{K}_p)^\top\end{bmatrix}}_{\boldsymbol{\Pi}}\boldsymbol{\Theta} = \begin{bmatrix}-\mathrm{vec}(\boldsymbol{A}^\top\boldsymbol{P}_i + \boldsymbol{P}_i\boldsymbol{A}) \\ [(\boldsymbol{X}^d-\boldsymbol{X})^\top \otimes (\boldsymbol{X}^d-\boldsymbol{X})^\top]^\dagger\boldsymbol{\Xi}^\top\end{bmatrix}. \tag{37}$$

Notice that matrix $\boldsymbol{\Pi} \in \mathbb{R}^{2n^2\times(n^2+m^2)}$ is a full rank matrix whose rank is obtained by $\mathrm{rank}(\boldsymbol{\Pi}) = \min(2n^2, n^2+m^2)$. This set of constraints ensure that $\boldsymbol{Q}_i$ and $\boldsymbol{R}_i$ converges in the limit to the real matrices $\boldsymbol{Q}_p$ and $\boldsymbol{R}_p$, i.e, $\lim_{i\to\infty}\boldsymbol{Q}_i = \boldsymbol{Q}_p$ and $\lim_{i\to\infty}\boldsymbol{R}_i = \boldsymbol{R}_p$.

Supplementary Note 6.5 Additional results

We conduct simulation studies using a linearized model of the drone dynamics around the hover flight condition. This model is valid under small angle condition. We design a continuous-time LQR, under

the following weight matrices $\boldsymbol{Q} = \text{diag}\{1,1,1,5,5,5\}$ and $\boldsymbol{R} = 5\boldsymbol{I}_3$, to guarantee trajectory tracking and fulfil the small angle condition. The optimal control gain is

$$\boldsymbol{K} = \begin{bmatrix} 0 & 0.4472 & 0 & 0 & 1.0446 & 0 \\ -0.4472 & 0 & 0 & -1.0446 & 0 & 0 \\ 0 & 0 & 0.4472 & 0 & 0 & 1.1907 \end{bmatrix}.$$

The problem is reduced to estimate the weight matrices associated to the quadratic reward function. The proposed model-based IRL is trained in 5,000 episodes with an arbitrary stabilizing control policy. The inferred weight matrices are

$$\boldsymbol{Q}_{5000} = \begin{bmatrix} 1.0004 & 0 & 0 & 0.0009 & 0 & 0 \\ 0 & 1.0004 & 0 & 0 & 0.0009 & 0 \\ 0 & 0 & 1.0004 & 0 & 0 & 0.0011 \\ 0.0009 & 0 & 0 & 5.0022 & 0 & 0 \\ 0 & 0.0009 & 0 & 0 & 5.0022 & 0 \\ 0 & 0 & 0.0011 & 0 & 0 & 5.0028 \end{bmatrix}, \quad \boldsymbol{R}_{5000} = 4.998\boldsymbol{I}_3$$

$$\boldsymbol{K}_{5000} = \begin{bmatrix} 0 & 0.4474 & 0 & 0 & 1.0450 & 0 \\ -0.4474 & 0 & 0 & -1.0450 & 0 & 0 \\ 0 & 0 & 0.4474 & 0 & 0 & 1.1911 \end{bmatrix}.$$

Here, the values of the reward function give a constraint to the algorithm to converge approximately to their exact values in the short term. The inferred matrices converge to their real values in the long term, i.e., when $i \to \infty$. We further verify the approach for the following non-diagonal positive definite weight matrix $\boldsymbol{Q}$ and diagonal matrix $\boldsymbol{R}$

$$\boldsymbol{Q} = \begin{bmatrix} 2.7566 & -1.1459 & 0.0697 & -1.2528 & 1.1637 & 0.0510 \\ -1.1459 & 0.9131 & 0.4855 & 0.3383 & 0.4985 & -0.1989 \\ 0.0697 & 0.4855 & 2.9506 & -1.1975 & 0.9104 & -2.3956 \\ -1.2528 & 0.3383 & -1.1975 & 4.7690 & -1.4049 & 1.9434 \\ 1.1637 & 0.4985 & 0.9104 & -1.4049 & 6.4813 & 0.5853 \\ 0.0510 & -0.1989 & -2.3956 & 1.9434 & 0.5853 & 3.4602 \end{bmatrix}, \quad \boldsymbol{R} = 10\boldsymbol{I}_3.$$

The optimal control gain is

$$\boldsymbol{K} = \begin{bmatrix} -0.0448 & 0.2217 & 0.1314 & -0.1056 & 0.8256 & 0.1114 \\ -0.5225 & 0.2020 & 0.0005 & -0.7548 & 0.1056 & -0.2299 \\ 0.0249 & 0.0366 & 0.5270 & 0.0502 & 0.0243 & 0.8792 \end{bmatrix}.$$

The inferred matrices for 5,000 episodes are

$$\boldsymbol{Q}_{5000} = \begin{bmatrix} 2.7577 & -1.1463 & 0.0697 & -1.2512 & 1.1634 & 0.0515 \\ -1.1463 & 0.9134 & 0.4857 & 0.3376 & 0.4993 & -0.1989 \\ 0.0697 & 0.4857 & 2.9517 & -1.1975 & 0.9109 & -2.3936 \\ -1.2512 & 0.3376 & -1.1975 & 4.7713 & -1.4056 & 1.9442 \\ 1.1634 & 0.4993 & 0.9109 & -1.4056 & 6.4840 & 0.5857 \\ 0.0515 & -0.1989 & -2.3936 & 1.9442 & 0.5857 & 3.4636 \end{bmatrix}, \quad \boldsymbol{R}_{5000} = 9.996\boldsymbol{I}_3$$

$$\boldsymbol{K}_{5000} = \begin{bmatrix} -0.0448 & 0.2218 & 0.1315 & -0.1056 & 0.8259 & 0.1114 \\ -0.5227 & 0.2021 & 0.0005 & -0.7551 & 0.1056 & -0.2300 \\ 0.0249 & 0.0367 & 0.5273 & 0.0502 & 0.0243 & 0.8796 \end{bmatrix}.$$

The numerical results verify that the proposed IRL is capable to infer the reward function for different weight matrices.

Supplementary References

[1] M. Street, "Drone identification and tracking," 2021. Available at https://kaggle.com/competitions/icmcis-drone-tracking.

[2] J. Whelan, T. Sangarapillai, O. Minawi, A. Almehmadi, and K. El-Khatib, “Uav attack dataset,” 2020. Available at https://dx.doi.org/10.21227/00dg-0d12.

[3] A. Keipour, M. Mousaei, and S. Scherer, “Alfa: A dataset for UAV fault and anomaly detection,” *The International Journal of Robotics Research*, vol. 40, no. 2-3, pp. 515–520, 2021.

[4] T. Rodrigues, J. Patrikar, *et al.*, “Data collected with package delivery quadcopter drone,” *Carnegie Mellon University*, pp. 1–15, 2020.

[5] D. Last, P. Thomas, S. Hiscocks, J. Barr, D. Kirkland, M. Rashid, S. B. Li, and L. Vladimirov, “Stone soup: announcement of beta release of an open-source framework for tracking and state estimation,” in *Signal Processing, Sensor/Information Fusion, and Target Recognition XXVIII*, vol. 11018, pp. 52–63, SPIE, 2019.

[6] Y. Xiao and X. Zhang, “Micro-UAV detection and identification based on radio frequency signature,” in *2019 6th International Conference on Systems and Informatics (ICSAI)*, pp. 1056–1062, IEEE, 2019.

[7] A. M. Abdelhameed, H. G. Daoud, and M. Bayoumi, “Deep convolutional bidirectional LSTM recurrent neural network for epileptic seizure detection,” in *2018 16th IEEE International New Circuits and Systems Conference (NEWCAS)*, pp. 139–143, IEEE, 2018.

[8] G. Liu and J. Guo, “Bidirectional LSTM with attention mechanism and convolutional layer for text classification,” *Neurocomputing*, vol. 337, pp. 325–338, 2019.

[9] Z. Che, S. Purushotham, K. Cho, D. Sontag, and Y. Liu, “Recurrent neural networks for multivariate time series with missing values,” *Scientific reports*, vol. 8, no. 1, p. 6085, 2018.

[10] A. Mehdy and H. Mehrpouyan, “A multi-input multi-output transformer-based hybrid neural network for multi-class privacy disclosure detection,” *arXiv preprint arXiv:2108.08483*, 2021.

[11] F. M. Bianchi, S. Scardapane, S. Løkse, and R. Jenssen, “Reservoir computing approaches for representation and classification of multivariate time series,” *IEEE transactions on neural networks and learning systems*, vol. 32, no. 5, pp. 2169–2179, 2020.

[12] G. E. Karniadakis, I. G. Kevrekidis, L. Lu, P. Perdikaris, S. Wang, and L. Yang, “Physics-informed machine learning,” *Nature Reviews Physics*, vol. 3, no. 6, pp. 422–440, 2021.

[13] K. Fujiwara *et al.*, “Reservoir splitting method for eeg-based emotion recognition,” in *2023 11th International Winter Conference on Brain-Computer Interface (BCI)*, pp. 1–5, IEEE, 2023.

[14] A. Perrusquía and W. Yu, “Neural $\mathcal{H}_2$ control using continuous-time reinforcement learning,” *IEEE Transactions on Cybernetics*, vol. 52, no. 6, pp. 4485–4494, 2022.

[15] A. Perrusquía and W. Yu, “Identification and optimal control of nonlinear systems using recurrent neural networks and reinforcement learning: An overview,” *Neurocomputing*, vol. 438, pp. 145–154, 2021.

[16] A. Perrusquía, R. Garrido, and W. Yu, “Stable robot manipulator parameter identification: A closed-loop input error approach,” *Automatica*, vol. 141, p. 110294, 2022.

[17] C. Sun, M. Song, S. Hong, and H. Li, “A review of designs and applications of echo state networks,” *arXiv preprint arXiv:2012.02974*, 2020.

[18] P. J. Baddoo, B. Herrmann, B. J. McKeon, J. Nathan Kutz, and S. L. Brunton, “Physics-informed dynamic mode decomposition,” *Proceedings of the Royal Society A*, vol. 479, no. 2271, p. 20220576, 2023.

[19] E. Weinan, “A proposal on machine learning via dynamical systems,” *Communications in Mathematics and Statistics*, vol. 1, no. 5, pp. 1–11, 2017.

[20] W. Xue, P. Kolaric, J. Fan, B. Lian, T. Chai, and F. L. Lewis, "Inverse reinforcement learning in tracking control based on inverse optimal control," *IEEE Transactions on Cybernetics*, vol. 52, no. 10, pp. 10570–10581, 2021.

[21] K. G. Vamvoudakis, "Q-learning for continuous-time linear systems: A model-free infinite horizon optimal control approach," *Systems & Control Letters*, vol. 100, pp. 14–20, 2017.
